# ‘The development sector is a graveyard of pilot projects!’ Six critical actions for externally funded implementers to foster scale-up of maternal and newborn health innovations in low and middle-income countries

**DOI:** 10.1186/s12992-018-0389-y

**Published:** 2018-07-27

**Authors:** Neil Spicer, Yashua Alkali Hamza, Della Berhanu, Meenakshi Gautham, Joanna Schellenberg, Feker Tadesse, Nasir Umar, Deepthi Wickremasinghe

**Affiliations:** 1 London School of Hygiene & Tropical Medicine, 15-17 Tavistock Place, London, UK; 2 Childcare and Wellness Clinics, 7 Fez Street, off Kumasi Crescent, Wuse II, Abuja, Nigeria; 3 Ethiopian Public Health Institute, P.O. Box 1242, Addis Ababa, Ethiopia; 4 London School of Hygiene & Tropical Medicine, B-140, Sushant Lok Phase 3, Gurgaon, 122011 Haryana India; 5 Independent Consultant, PO Box 30652, Addis Ababa, Ethiopia; 6 PACT, House 5, 16 PO WMafemi Crescent, Utako, Abuja, Nigeria

**Keywords:** Northeast Nigeria, Ethiopia, Uttar Pradesh, India, Scale-up, Maternal and newborn health, Innovations

## Abstract

**Background:**

Donors often fund projects that develop innovative practices in low and middle-income countries, hoping recipient governments will adopt and scale them within existing systems and programmes. Such innovations frequently end when project funding ends, limiting longer term potential in countries with weak health systems and pressing health needs. This paper aims to identify critical actions for externally funded project implementers to enable scale-up of maternal and newborn child health innovations originally funded by the Bill & Melinda Gates Foundation (‘the foundation’), or influenced by innovations that were originally funded by the foundation in three low-income settings: Ethiopia, the state of Uttar Pradesh in India and northeast Nigeria. We define scale-up as the adoption of donor-funded innovations beyond their original project settings and time periods.

**Methods:**

We conducted 71 in-depth, semi-structured interviews with representatives from government, donors and other development partner agencies, donor-funded implementers including frontline providers, research organisations and professional associations. We explored three case study maternal and newborn innovations. Selection criteria were: a) innovations originally funded by the Bill & Melinda Gates Foundation (‘the foundation’), or influenced by innovations that were originally funded by the foundation; b) innovations for which a decision to scale-up had been made, allowing us to reflect on the factors influencing those decisions; c) innovations with increased geographical reach, benefitting a greater number of people, beyond districts where foundation-funded implementers were active. Our data were analysed based on a common analytic framework to aid cross-country comparisons.

**Results:**

Based on study respondents’ accounts, we identified six critical steps that donor-funded implementers had taken to enable the adoption of maternal and newborn health innovations at scale: designing innovations for scale; generating evidence to influence and inform scale-up; harnessing the support of powerful individuals; being prepared for scale-up and responsive to change; ensuring continuity by being part of the transition to scale; and embracing the aid effectiveness principles of country ownership, alignment and harmonisation.

**Conclusions:**

Six critical actions identified in this study were associated with adopting and scaling maternal and newborn health innovations. However, scale-up is unpredictable and depends on factors outside implementers’ control.

## Background

The development sector is dominated by donor-funded health projects and programmes implemented by nongovernmental organisations that aim to develop innovative practices. It is hoped that these will be adopted by low and middle-income governments and scaled within existing health systems and programmes. Indeed, scale-up of donor funded projects is an important aim: it allows the geographical reach to be expanded to benefit a larger number of people, as well as having longer term benefits beyond time limited project periods. However, as the quote in our title captures, such innovations commonly end when project funding stops, thereby limiting any longer term and larger scale health impacts in countries with weak health systems and pressing health needs [17, 20, 23, 25, 28, 30]. This paper’s aim is to identify the critical actions for externally funded project implementers to enable scale-up, focussing on maternal and newborn child health innovations in three low-income settings.

The factors influencing whether innovative technologies and practices are adopted and scaled-up are widely reported in the literature. These include the attributes of the innovation itself, such as its simplicity, appropriateness for users and beneficiary communities, and whether it is adaptable across different contexts. Potential adopters’ attitudes, needs, knowledge and skills affect their ability and willingness to accept innovations, and policy champions and community opinion leaders often influence the adoption of innovations by government, health workers and beneficiaries [3, 4, 10, 12, 14, 23–25, 27–30]. Aspects of the contextual environments in countries where innovations are introduced also have a bearing on whether scale-up is possible or likely, including economic resources and their distribution, policy leadership and priorities, political ideologies and systems of governance, macroeconomic policies, issues of conflict and security and the absorptive capacity of health systems. Other factors include socioeconomic and cultural contexts such as religious institutions and ideas, hierarchical gender relations, and prevailing health beliefs and practices [1, 3, 14, 15, 22, 24]. Some studies identify actions that enable scale-up to happen including: implementers creating a scale-up plan and designing innovations that are scalable within the receiving environment; invoking the support of policy champions and opinion leaders; generating and effectively communicating evidence; effectively advocating government, while ensuring government’s close engagement in the design and development of an innovation; and increasing the capacity of the receiving organisation to implement at scale [4, 7, 25, 27].

While there is an extensive literature on scale-up, few studies use empirical evidence on the scale-up of innovative practices and technologies in low and middle-income countries. While some studies highlight actions that enable scale-up, the most important actions are not always identified. To address this gap, we conducted a qualitative study focusing on three Bill & Melinda Gates Foundation-funded and scaled maternal and newborn health innovations as in-depth case studies.

We defined maternal and newborn health innovations as *donor-funded interventions aiming to improve the survival of mothers and babies that are* ***new to the contexts that they are introduced and hence innovative****,* and that strengthen existing health systems through improving healthcare accessibility or the performance of health workers, and target poor, rural populations. We defined scale-up as: *the adoption and implementation of donor-funded maternal and newborn health innovations, thereby increasing their geographical reach to benefit a greater number of people beyond donor-funded implementers’ project districts or in the longer term beyond donor-funded project periods.*

The study was conducted in settings with some of the world’s highest burdens of maternal and neonatal mortality: Ethiopia, northeast Nigeria, and the state of Uttar Pradesh in India. Table 1 provides basic maternal and newborn health indicators for each setting. One scaled innovation was selected from each setting for in-depth study. This enabled us to address the aim of this paper: to identify the critical actions adopted by donor-funded implementers that enabled their maternal and newborn health innovations to be scaled by host country governments. Despite the focus on maternal and newborn health innovations, the list of critical actions may be of value to implementers, donors and other stakeholders in planning and developing health innovations more generally in low- and middle-income countries.

**Table 1:** Maternal and newborn health indicators in Ethiopia, Nigeria and Uttar Pradesh

Setting	Maternal mortality rate per 100,000 live births	Neonatal mortality rate per 1000 live births	Year
Ethiopia	420	28	2015
Nigeria	560	34	2015
Uttar Pradesh	440	45	2011

Sources: [8]; [9]; [26]

## Methods

### Case studies

We adopted a case study approach [31]. Our case studies were Bill & Melinda Gates Foundation-funded maternal and newborn health innovations that had been scaled-up. Selection criteria were: a) they were originally funded by the Bill & Melinda Gates Foundation (‘the foundation’), or influenced by innovations that were originally funded by the foundation; b) the decision had been made to scale up, allowing reflection on the factors influencing those decisions; c) their geographical reach was increased to benefit a greater number of people beyond districts where foundation-funded implementers were active. Based on these criteria we selected three case studies from among all maternal and newborn health innovations funded at the time by the foundation in the study settings. We further distinguished between: a) ‘government-led scale-up’, meaning country and state governments deciding on, adopting, funding and leading on the implementation of maternal and newborn health innovations at scale while benefiting from support and learning from donor-funded pilot innovations; and b) ‘donor-led scale-up’ meaning other donors deciding on, adopting and funding donor-funded innovations at scale. Our three case studies were:

### Government-led scale-up

#### Sepsis case management in Ethiopia

An innovation allowing community health workers (Health Extension Workers - HEWs) to administer antibiotics to newborn babies with bacterial infections where referral to health facilities was not possible, in 19 districts, implemented by Save the Children USA with foundation funding. The intended outcome was to expand access to effective newborn sepsis case management. The innovation was scaled as one of nine components of the government-led flagship programme *Community Based Newborn Care,* in 92 districts over a first phase and implemented by a consortium of international nongovernmental organisations. The Ethiopian Ministry of Health financed the programme with contributions from multiple external donors.

#### *mSehat* in Uttar Pradesh, India

An innovation that involved supplying smart phones with an application (app) for community health workers (Accredited Social Health Activists - ASHAs and Auxiliary Nurse Midwives) with features to facilitate health communication, workflow checklists and a patient registration system as part of a wider maternal and newborn health data platform. The outcomes of the innovation were expected to include more effective interactions between ASHAs and communities. The innovation was implemented between 2015 and 2017 by a private provider as part of a public private partnership in five of the 75 districts in Uttar Pradesh, with three years of funding from a state government agency. It was influenced by multiple small-scale (typically in one district) ‘proof of concept’ mobile phone-related innovations in the states of Uttar Pradesh and Bihar, funded by external donors including the foundation. The innovation, implemented as it was in five districts, was considered by the Government of Uttar Pradesh to be an ‘expanded pilot’ for potential scale-up across the state.

### Donor-led scale-up

#### *Emergency Transport Scheme* (ETS) in Northeast Nigeria

An innovation to incentivise taxi drivers to transport women to health facilities for childbirth implemented across Gombe State by Society for Family Health, a Nigerian nongovernmental organisation, and Transaid, a UK-based nongovernmental organisation, with foundation funding. The outcomes of the innovation were expected to be improved accessibility to health facilities among pregnant women. The innovation was scaled with some modifications across the neighbouring Adamawa State with three years of funding from the UK-based charity Comic Relief, implemented by Transaid, with support from Society for Family Health.

### Research questions

The study focused on identifying the critical actions that implementers had adopted to catalyse scale-up of these three case study maternal and newborn health innovations. Based on the scale-up literature (including [4, 7, 24, 25, 27]) we explored the following themes:

- How the case study innovations were designed to be scalable;
- Implementers’ actions to influence decisions to scale those innovations, including whether and how evidence was used to influence scale-up and the role of key actors in shaping the decision;
- Ways implementers contributed to and supported the implementation of the scaled innovations;
- Contextual factors influencing scale-up relating to policy decision making and implementation at scale.

### Data collection and analysis

A common topic guide was drafted to allow for direct comparison across the three case studies and settings. Researchers from Ethiopia, Nigeria, India and the UK reviewed the guide and made adaptations to reflect different country contexts. It was then used as the basis of semi-structured interviews with stakeholders in the field of maternal and newborn health, who were purposively selected for having detailed knowledge of the scale-up of the case study innovations. We selected semi-structured interviewing as our method of data collection since this approach is appropriate for capturing experiences, perspectives, practices and processes [6]. A total of 72 interviews were conducted across the three settings between March 2014 and December 2015. Our respondents represented different constituencies: national and sub-national government, development partners including donors and UN agencies, civil society and private sector implementers of the case study innovations, stakeholders from professional associations and research organisations, as well as frontline implementers delivering the innovations – namely HEWs, ASHAs and taxi drivers (Table 2). This selection of allowed us to capture a balance of views from the different constituencies. The respondents included: managers and directors, technical advisors, programme officers, and evaluation officers and researchers. The interviews were conducted by researchers with substantial experience of qualitative methods. A deductive and inductive approach was taken; we both examined a priori themes and were attentive to and explored emerging themes in our interviews. Our interviewing was consistent with widely accepted approaches to maintaining validity in qualitative methods, including posing open questions rather than leading questions, posing broader questions before more specific probes, and as discussed below, we used triangulation approaches to cross check our findings [19].

**Table 2:** Interviews by type of organisation

Type of organisation	Number of interviews
Implementer organisations	40
Government	14
Development partners including donors and multilateral organisations	11
Researchers, technical experts and members of professional associations	7

To preserve confidentiality, interviews took place in private spaces, and all respondents gave informed consent before each interview. We used digital sound recorders for data capture where respondents agreed. The data were analysed based on the framework approach [18]. Interviewers wrote up expanded field notes shortly after each interview, based on sound recordings and comprising detailed notes and direct quotes organised under analytic headings. These represented both a priori and emerging themes, as suggested by [13]. Hence, by simultaneously capturing and analysing data under thematic headings, we could identify emerging interpretations and themes for exploration in subsequent interviews. We then created a common analytic framework enabling us to identify and compare common themes across the three settings, as well as those that were specific to individual case studies and contexts [11]. Based on the analytic framework NS and DW coded the expanded field notes. NS drafted the paper, which was critically reviewed by all authors.

We adopted several methods to enhance the validity of our findings [19]. We triangulated our data; stakeholders were interviewed from multiple organisations and we cross-verified their accounts enabling us to form a balanced interpretation of the issues being explored. An investigator triangulation approach was taken where we compared and agreed different researchers’ analyses; hence the findings presented in this paper are the interpretation of multiple researchers. We also conducted member checks by presenting and discussing emerging findings with stakeholders in Addis Ababa, Lucknow, London, Seattle and Vancouver. There was substantial consistency between different stakeholder groups on the issues we explored. Our results reflect the views from the different stakeholder groups we interviewed: we present common views across the full range of respondents rather than a select few.

## Results

Our respondents spoke about the importance of scaling donor-financed innovations to maximise longer term value from what were substantial donor investments, but accepted that innovations were commonly not scaled beyond their original project periods. Speaking about Nigeria, a development partner lamented: *‘If a project dies after funding, for me it is not a successful project…’;* while in India a government official exclaimed: *‘The development sector is a graveyard of pilot projects!’* An enduring problem reported by our respondents was that short project periods of three to four years limited what could be achieved, which could be wasteful and created uncertainty for both implementers and governments. One government respondent in India captured this point: *‘…any donor funds should be at least six or seven years...because when you fund a four-year project…one year goes on recruitment and then the last six months is winding up…’.* Nevertheless, as our three case studies show, scale-up of innovations that are part of short-term projects is possible. Based on our qualitative data we identified six critical actions adopted by implementers that were vital to the scale-up of one or more of our three case study innovations. These were:

1. Designing innovations for scale;
2. Generating evidence to influence and inform scale-up;
3. Harnessing the power of individuals;
4. Being prepared and responsive;
5. Ensuring continuity;
6. Embracing the principles of aid effectiveness.

The following sections explore each of these critical actions in turn.

### Designing innovations for scale

The first critical action reported by our respondents was that scale-up had been integral to the design of our case study innovations: *‘...if you plan scale-up when your pilot’s over there are many things you can’t go back and correct…’* a government respondent from India explained. Some stakeholders pointed out that despite this happening, it was challenging to develop innovations that were scalable; indeed, their experimental nature carried the inherent risk that they would not be effective. Hence, despite putting substantial energy into the process of designing, testing and adapting innovations for scale, externally funded innovations commonly fail. A development partner reflected: *‘If you are lucky, that process will generate some… innovations that can be moved to scale...designing and testing and adapting only spits out a sample of innovations that are truly scalable if you are lucky...’*. There were several common attributes of the case study innovations that respondents described as important to their scalability – these are listed as follows.

#### Perceived as effective

Our respondents reported that it was vital that the case innovations had been perceived as effective. In Ethiopia, randomised control trial evidence suggested that the sepsis case management innovation had impacted on health outcomes, that justified its scale-up: *‘It was taken because it produced results…,’* said an implementer. In Uttar Pradesh, government and civil society respondents noted that while evidence of quantitative impacts on health outcomes was not generated, our respondents reported that mSehat had demonstrably helped frontline workers to fulfil their roles more effectively*.* Impact data were also not available in Nigeria, however: *‘Because the project has such a widespread history…it was kind of like easy… to demonstrate effectiveness and impact,’* an implementer suggested.

#### Required modest resource inputs

Respondents explained that the innovations required financial resources that the government could afford and modest human resource inputs; this was essential for governments, with finite budgets and many competing priorities, to consider them for scale. Modest financial costs were important for ETS in Nigeria, where respondents were clear that paying drivers would not work: *‘...the programme is fairly sustainable…because drivers are giving up their time voluntarily’.* mSehat was based on a low-specification, relatively low-cost handset and ASHA training was short; an implementer observed: *‘…it’s feasible to train them on this and it doesn’t take an entire lifetime to learn...’.* Similarly, sepsis case management in Ethiopia was based on a short training period, which respondents explained was a very important aspect of its scalability. However, it had been difficult for implementers to balance committing substantial effort and resources to ensure the success of their innovations, with creating innovations that were not resource-intensive, and therefore scalable with limited government budgets. A government official in Uttar Pradesh summarised: *‘…at limited scale you can do anything – but we deliberately avoided doing such things that’ll not be possible to scale-up...’.*

#### Acceptable to and incentivised frontline workers

Respondents pointed to the ways the innovations were considered acceptable to frontline workers and thereby incentivised their adoption. In Uttar Pradesh, mSehat’s user interface was designed to be easily understood by ASHAs, as one implementer explained: *‘…even those that have minimum qualifications will face no problem in adequately using the application once they get used to it through our training programmes’.* ASHAs would also receive material incentives; entering data electronically meant faster performance-based payments than the former paper-based system, and the innovation reduced their workloads: their tasks such as counselling could be performed more quickly and meant they no longer carried heavy logbooks and other materials. Similarly, in Nigeria, the innovation incentivised taxi drivers involved in the scheme by allowing them to park their vehicles at the front of passenger loading queues, although it was accepted that this had been less effective in remoter areas with few taxis. In the three settings, goodwill and satisfaction incentivised frontline workers. Our respondents explained that in Ethiopia HEWs gained satisfaction by saving babies lives, which increased their acceptance and credibility within the community. Likewise, the innovation gave ASHAs in Uttar Pradesh more confidence to work among communities, and in northeast Nigeria branded tee shirts, caps and car stickers engendered feelings of pride and commitment. *‘We believe that God Almighty is going to reward all of us; that’s why many of us will not even collect money,’* said a driver. The ETS scheme had improved drivers’ standing among communities; a driver revealed: *‘…people see us as useless, good-for-nothing rascals…this programme’s an opportunity for us to change that image…’.* The smart phone innovation in Uttar Pradesh was also described as aspirational, and as having novelty value: an implementer said: *‘…of course the novelty factor. Hey! We are doing something new and get a smartphone…’.*

#### Acceptable to communities

The innovations met communities’ needs, were culturally acceptable and aligned with local cultural and traditional practices. Capturing this point, an implementer in Ethiopia remarked: *‘The community has to accept it and say, “this is actually something that can solve my problems”’*, while in Nigeria a professional association representative said: *‘If the community does not accept you then there is no way you will be able to scale-up’.* Ascertaining innovation acceptability depended on conducting formative research with community leaders, and the innovations were designed accordingly; for example, our respondents reported that the use of locally relevant visual and audio material for the mobile phone app in Uttar Pradesh was very important to its acceptance by communities.

#### Adaptable across diverse geographical contexts

The innovations were described by our respondents as being adaptable across diverse sociocultural contexts and to variations in the strength of the health system between different areas; an implementer summed up: *‘We keep saying it’s not one size fits all – in a country like Ethiopia we have to have different approaches and strategies in different areas’.* Nevertheless, many features of the three innovations were also standardised, which made them cost effective at scale: *‘There might be some innovative variations…but in terms of...training, supervision, it’s all quite standardised’,* clarified a development partner in Ethiopia. Similarly, in Nigeria, while there was flexibility to adapt to diverse geographical settings, *‘…the principles of the scheme have to remain the same...’*, one implementer explained.

### Generating evidence to influence and inform scale-up

A second critical action relates to evidence. In the three settings, our respondents reported that quantitative survey data establishing an innovation’s impacts on health outcomes, while important, had not been a critical part of decisions to scale innovations. Instead, first-hand, experiential evidence fostered emotional buy-in from government and other decision makers and influenced decisions to scale innovations. In addition, qualitative evidence documenting implementation processes, together with syntheses of secondary data, showed which innovations were feasible in practice and offered lessons about how they could be implemented at scale.

In Ethiopia, quantitative evidence of impacts on health outcomes generated by the implementer and periodically shared with the government had been necessary. However, this evidence had helped justify, rather than trigger, the decision, which was made before the end-line quantitative survey results were released. A development partner explained: *‘The policy breakthrough is never the data, the findings themselves...it’s the trust, the relevance, it’s being at the table, being able to show you support implementation...’.* Indeed, first-hand evidence in the form of project visits was considered very influential: *‘We took [government decision makers] out, we showed them…that was very convincing to them...’,* said an implementer in Ethiopia. Evidence of impacts did not influence the funder’s decision to scale ETS in Nigeria. Indeed, it had been difficult to collect such evidence because drivers were often unwilling to keep activity records, and some were illiterate. An implementer explained: *‘The driver’s responsibility is to get the woman to the health facility and often they go beyond the call of duty…when it comes to us pestering them for data sometimes they don’t see it as a priority’.* Instead, the project’s plausibility compensated for the lack of data; a researcher said: *‘…who can resist saving mothers and babies’ lives? It’s a fantastically plausible project…’*. Similarly, in Uttar Pradesh, evidence of health impacts did not influence the decision to finance mSehat. Instead, it was inspired by the fact that externally funded implementers had demonstrated that mobile phone-based innovations were implementable in India. An implementer reflected: ‘*My experience in [Uttar Pradesh] has been more than the data, more than the impact [it’s about]…what’s happening in the field...they want to have a taste of it...’*

Respondents in the three settings reported that implementers had collected qualitative evidence documenting implementation experiences and challenges, and that this evidence had been important in helping to scale the innovations effectively. In Ethiopia, such evidence showed the best ways to train HEWs to identify danger signs and administer antibiotics to sick babies, demonstrated that they could correctly refer more complex cases and that communities accepted this. While generating ‘local’ qualitative evidence was considered by our respondents to be vital, the implementer also synthesised evidence from other countries. This engendered acceptance by Ethiopia’s government and helped design the scaled innovation: *‘Save the Children’s role was…in bringing out other countries’ evidence and…making sure we have included the key interventions and followed already tested and feasible strategies,’* explained an implementer.

In Uttar Pradesh, mSehat adopted lessons from the multiple donor-funded pilot projects: *‘[mSehat tried to] absorb what was there in the field...all of these [pilot innovations] shaped it…,’* a development partner reflected. In Nigeria, lessons learned from implementing the innovation in Gombe State, including ways to increase driver retention, were important in informing the development of the innovation in Adamawa State. A development partner said: ‘*…between all those different pieces of learning, even if they weren’t externally validated…some learning had been done’.*

### Harnessing the power of individuals

A third critical action to catalyse scale-up was implementers mobilising the support of policy champions perceived to have power, connections and influence at high levels from government and development partner agencies as well as traditional rulers, who could convince others that an innovation should be scaled. In the three settings, personal connections with government decision makers were described as being as, if not more, important than formally engaging government: *‘Having some experience of working in Nigeria it’s very important to engage on the personal level...,’* said an implementer. However, while building and maintaining relationships with powerful individuals had been vital, respondents acknowledged that with so much depending on this approach there was a risk that if that person left they would need to elicit support afresh.

In Uttar Pradesh, there was strong interest among key senior state government officials in using digital technology to address health needs in rural areas, including the Chief Minister, *‘…who very much believed that technology was the solution to many of the problems in administration…that was a major factor which paved the way for scale-up’,* according to a government official. One particular government champion was reported as critical, as an implementer clarified: *‘…because he’s a renowned policymaker his words are looked up to with respect, so that helped us a lot. If you ask me any single thing…I think it’s [this person’s] vision and passion and belief - one [person] can make a difference!’* Similarly, in Ethiopia, a senior health official’s support was crucial to government agreeing to scale the innovation: *‘Convincing him, identifying him as a champion...that was very, very decisive’*, an implementer explained. While in Nigeria the innovation was not scaled with government funding, seeking the acceptance and endorsement of senior government officials within Adamawa State was an essential part of introducing the innovation there; an implementer summarised: *‘The state [Commissioner for] Health was quite pivotal in allowing the programme to progress further’.*

In Ethiopia, individuals from development agencies also contributed to the decision to scale the innovation; *‘What was the magic in Ethiopia? It does come down to some of the personalities!’* said a development partner, who also exclaimed that the commitment and belief of one individual had been vital: *‘[This person]…had a really good relationship with the Ministry of Health...and was a really proactive person. [Their] personality was definitely a factor...’.* In Nigeria, traditional rulers were influential. Although some rulers were supportive of the introduction of innovations, many rulers embracing conservative views had resisted externally funded health programmes, as was the case in Adamawa State. Hence, the implementer had not engaged them there.

### Being prepared and responsive

A fourth critical action was implementers both ensuring that scale-up was integral to their project plans and being responsive to change. The former involved conducting formative research to assess policy, health systems, geographical and sociocultural contexts to help to design innovations that were culturally appropriate and aligned with country health policies, programmes and targets. A development partner speaking about Nigeria summarised: *‘For scale-up to occur successfully we cannot rely on luck… if we go to the field without a prepared agenda, we’ve set ourselves up for failure’*. Formative research also formed the basis of implementers’ advocacy plans, including identifying which actors they could work with and those they needed to petition for support. In Nigeria, formative research was important to ascertain which districts were sufficiently safe to implement the innovation in the context of the security situation.

While preparing for and anticipating scale-up had been important, policy contexts were not static in the three settings; changes in government administrations following elections in Nigeria and Uttar Pradesh inevitably led to changes in policy direction. As our respondents pointed out, implementers had to be responsive to change over the course of their project’s lifespan to remain relevant. A development partner in India explained: *‘Flexibility is key to go apace with government; anything that you work on, you have to keep their struggles, timelines, and accordingly adjust yours. You have to be patient for this’.* In Ethiopia too, respondents stressed that health policy ideas changed quite often and hence it was important it was for donors and implementers to be responsive to the government’s thinking; an implementer said: ‘*Knowing when to push your agenda and when to back off is really important’.* Civil society implementers, in particular, needed to ensure they worked around the Ethiopian government’s agenda: *‘[It’s] the tricky role nongovernmental organisations have to balance in Ethiopia,’* one development partner revealed. Respondents suggested that a policy window emerged in Ethiopia created by three factors: increased political support following a study visit to Nepal, which demonstrated that community-based sepsis case management was feasible; the release of national figures showing limited improvements in neonatal and maternal mortality; and increased government confidence that its Health Extension Program was sufficiently robust to support additional innovations. An implementer explained: *‘[Events came together] in a certain pivotal moment where the Ministry decided there’s going to be a policy shift...’* The implementer responded to this 'pivotal moment', as a development partner reflected: *‘They were flexible and nimble...recognising hey, there’s an opportunity here!’*

### Ensuring continuity in the transition to scale

The fifth critical action to catalyse scale-up was the importance of continuity of implementers’ involvement in implementing the scaled innovations rather than, as is common among many projects, their involvement stopping when project funding ran out. Across our three case studies, the externally funded implementers contributed to delivering the scaled innovations. Transaid acted as lead implementer in the scale-up State of Adamawa; Save the Children was part of an implementing consortium for the Community-Based Newborn Care programme in Ethiopia; and in Uttar Pradesh, Intrahealth, the implementer of a foundation-funded project, became part of the public private partnership implementing mSehat. Our respondents suggested that it was particularly important to harness the experience of project staff within the scaled programme; an implementer in Uttar Pradesh captured: *‘Who else in that consortium has any on the ground experience of these things? So obviously [the implementer] brings a lot of that to the consortium…’* An implementer in Nigeria summarised the value of harnessing experience and project materials within the scaled programme: *‘If you want to scale [the innovation], what are the lessons learned in the pilot…? What mistakes have we made, what challenges have we faced…? That initial pilot helped to serve as a guide’.*

In Ethiopia, study respondents reported that Save the Children had participated in workshops to develop and design the scaled innovation, including helping to draft implementation plans and guidelines, which also enabled them to influence government thinking. *‘...We used that as an opportunity to integrate as many newborn-care-related activities as possible in that implementation plan...,’* an implementer remarked. Helping to build the capacity of Ethiopian regional government staff and systems was also an important step to enabling the Government to implement the innovation at scale. An implementer said: ‘*The [health] system may not be ready. So, when you go for scale-up you have to put into your programme other components to strengthen the health system...’*

### Embracing aid effectiveness principles

The sixth critical action pointed to by our respondents was that the externally funded implementers had embraced the aid effectiveness principles of country ownership, whereby the government took ownership of the innovation; alignment, meaning externally funded innovations fitted closely with country programmes, priorities and targets; and harmonisation, that is, coordination among donors and externally funded implementers.

#### Country ownership

In Ethiopia and Uttar Pradesh, it was critical that government owned the innovations rather than them being presented as donor projects. The Ethiopian government expected donors and their implementers to support country policies and therefore there was considerable involvement and consultation by the government in the development of the sepsis case management innovation; one implementer summed up: *‘Government is always in the driving seat’*, while another implementer revealed:

*If you see how things work in Ethiopia, [development partner] staff almost work for the government. They support the Ministry at all levels…they don’t just sit in their offices and work on their own projects. They are mindful that they are supporting the national health system….*

Similarly, mSehat was presented as state government-led and owned rather than introduced by donors, although government respondents acknowledged that externally funded pilot innovations had encouraged the state government to finance mSehat, and that design features and learning from pilot projects had fed into the innovation’s design through extensive consultation between the state government and the implementers of the pilot projects. A development partner explained: *‘[The donor]…took a very small, critically important role – the triggering role. But now it’s government’s baby…’,* while a government official clarified: *‘Funding is from [government]…The intellectual property will be 100% owned by [government]; that’s something we really care about’.* Hence, in both settings, externally funded implementers closely engaged with government from inception and through the project’s life. In contrast, government ownership of ETS was limited, reflecting the fact that it was scaled in Adamawa state with donor, rather than government, funding. Indeed, our respondents explained that rural health programmes in northeast Nigeria were commonly donor financed, with state governments adopting a limited role in leading and coordinating externally funded programmes. Nevertheless, Transaid did closely engage with government actors to introduce the innovation: *‘…government officials were really happy about the programme because they said that they are ever ready to assist us if we need their help…,’* according to an implementer. Relationship building was therefore critical in the three settings; as suggested earlier, the support of individuals was vitally important. Thus, country ownership meant influential individuals in government having strong ownership of the innovations. In Ethiopia, an implementer remarked: *‘…ongoing informing and engagement was key for the government to really go ahead with the start of scale-up’.*

#### Alignment

Respondents across three settings agreed that our case study innovations closely fitted with country priorities, programmes and targets. While Ethiopia received substantial development assistance for health, aligning with government programmes was critical for donors and their implementers. Respondents agreed that the sepsis case management innovation closely corresponded with country health programmes and was acknowledged as helping to achieve the government’s under-five mortality targets; hence government agreed to its scale-up. One implementer explained: *‘…it addresses a major health problem and that problem is a government priority, so it aligns with that. [And] it builds on the system; it isn’t a parallel system’.* In Uttar Pradesh, reductions in donor budgets meant their roles were changing from funding agencies, with some influence on government, to providing technical assistance in support of government initiatives. An implementer said: *‘…it’s clearly not donors who are calling the shots here. They have to align with what the government wants.’* Hence, to influence the development of mSehat, pilot project implementers closely aligned their work with state government priorities. While in Nigeria the government did not finance the innovation at scale, the implementers did need to present the innovation as fitting with the Adamawa state government’s goals. As a development partner pointed out, the importance of presenting the work using the right terms helped align it with government thinking: *‘If we look at it as just ETS, then it doesn’t align with anything; if you call it [maternal and newborn child health] then it aligns with everything’*.

#### Harmonisation

Harmonisation was also an important factor that contributed to scale-up. In Ethiopia, harmonisation was improving, largely due to the government taking a strong coordination role through the government-led *National Child Survival Technical Working Group*. An implementer observed: *‘The Technical Working Group was one voice, not a single organisation or individual voice… it was able to leverage, at least within that group, a consensus.’* Engaging with this group had been important to government’s adoption of sepsis case management; it gave Save the Children a legitimate channel to communicate with government and importantly, to demonstrate its willingness to harmonise its efforts with other actors*.* In Uttar Pradesh and Nigeria, however, harmonisation was problematic. A government official in India outlined the reality: *‘Everybody will talk of…not working in silos and all that. But… if there are two different donors and there are two projects in the same area they will not share any information…’* Limited harmonisation meant implementers could not benefit from what was learned by others, and poorly coordinated communication with government, and indeed competition for government attention, made decision-making relating to scale-up difficult. A development partner acknowledged that coordination at the federal level in Nigeria was improving, although not so at state and district levels:

*When we sit over tea or coffee we say “yes”, and when we go back to our organisation we do our own things…we come with money and expertise and we confuse them with conflicting information…we disempower government and make them feel like puppets; we need to give them permission to be in charge because they are in charge, and we need to give them that power and support them.*

## Discussion

We identified six critical actions that donor-funded implementers adopted to foster scale-up: each action was critical to the scale-up of at least one of the case study innovations we explored:

1. They designed innovations for scale;
2. They generated a strong base of evidence to influence and inform scale-up;
3. They harnessed the support of powerful individuals;
4. They prepared for scale-up and were responsive to change;
5. They ensured continuity by being part of the transition to scale process;
6. They embraced the aid effectiveness principles of country ownership, alignment and harmonisation.

Most of these critical actions were common across our case studies, although there were differences reflecting very different country contexts. While each innovation was different, they shared several design attributes that promoted their scalability. They were perceived as effective, they were relatively simple and required modest resource inputs, they incentivised frontline workers to use them, they were acceptable and reflected cultural and traditional practices within the communities into which they were introduced (a point also made by [16]), and they were adaptable across diverse geographical contexts. Also in common was that quantitative evidence of impacts had not substantially influenced decisions to scale each of the case study innovations. Instead, other forms of evidence were important including qualitative evidence, secondary data and first hand experiential evidence. Having the support of powerful individuals had been critical to the scale-up of the three innovations: government officials in all settings, individuals from development agencies in Ethiopia and traditional rulers in northeast Nigeria. The fact that the implementers had prepared for scale-up was also important, as was their responsiveness to changes in country priorities and programmes, especially in Ethiopia where the implementer waited for a ‘pivotal moment’ to promote the innovation vocally. Also in common across the settings, the implementers each contributed to the implementation of innovations when they were being transitioned to scale, and in Ethiopia the implementer used their experience to help shape wider government strategy. The principles of aid effectiveness were presented by our respondents as important factors promoting scale-up. In all settings, our case study innovations closely aligned with country priorities, programmes and targets. Harmonisation among donors, development agencies and implementers was also significant, although it was only in Ethiopia where strong government coordination appeared to have contributed to the scale-up of the sepsis case management innovation. The governments of Ethiopia and Uttar Pradesh committed finances to scaling innovations and led on their implementation at scale; hence country ownership was critical in those places. These contrast with Nigeria, where despite efforts to advocate for government funding, the scaled innovation was implemented by a UK-based nongovernmental organisation based on a grant from a UK charity.

The existing scale-up literature points to the importance of designing innovations that are effective and scalable, the role of policy champions and the need to prepare for scale-up (for example, [4, 7, 25, 27]). Our study contributes a number of insights to the existing literature on scale-up. Our data show that these factors were also very important in three low-income settings with high burdens of maternal and neonatal mortality. Additionally, as our list of six actions highlights, scale-up depended on many factors in addition to the innovations being perceived as effective. A certain ‘magic’ was required consisting of good luck as well as having the support of powerful individuals, which was considered more important than formal decision making by organisations. It was also important that the implementers were able to respond when political and policy environments were favourable, as was the willingness of multiple donors and implementers to harmonise and align their activities, and the strong ownership of recipient governments, especially in Ethiopia. An important contribution of our study is that it highlights that while externally funded implementers can take steps to enable scale-up, scale-up can be unpredictable and dependent on many factors outside their control. Indeed, many innovations that are effective do not get scaled up; and while ineffective projects also do not get scaled-up, those with limited evidence of their effectiveness can be scaled, which was the case with our three example innovations [24, 25].

Another contribution of our study to existing literature on scale-up is that it focusses on project-based innovations. It seems likely that scale-up will remain difficult given the continued focus on short-term, project-based donor financing for nongovernmental organisations to implement pieces of work often falling outside country health strategies and programmes, budgeting cycles and monitoring and reporting systems [5, 21]. This mode of donor support has inevitably resulted in a multiplicity of parallel projects in Ethiopia, Uttar Pradesh and northeast Nigeria where many donors, other development partners and implementers working on health-related activities make harmonisation challenging. Harmonisation is often against implementers’ interests because they compete for donor funding and need to demonstrate to donors that impacts are attributable to their efforts. Poor harmonisation is problematic for governments with limited capacity to coordinate multiple externally funded activities, therefore making it difficult to make informed decisions about scale-up of donor-funded innovations. Project-based financing is also the embodiment of unpredictable aid – predictability being another widely recognised aid effectiveness principle [21]. Limited longer term predictability of funding heightens competition among implementers for short-term grants, which in turn undermines efforts at harmonisation [21, 25]. Short-term funding also limits what implementers can achieve, particularly when they aim to change ingrained cultural and traditional practices [2, 16]. Limited predictability is problematic for governments; it is difficult to forecast whether, when and how much funding donors will commit to standalone projects and hence whether and to what extent such projects might contribute to country targets and fit into long term strategic plans. Hence, recipient governments struggle to commit to funding and scaling externally funded innovations in the longer term. Yet, one justification for project-based working is that innovative ways of working can be tested. Donors, therefore, need to reflect on whether they can accept the probability that even projects deemed successful commonly end when project funding ends. While this approach – in the words of one of our respondents *‘…spits out a sample of innovations that are truly scalable if you are lucky...,’* it might be argued this is an inefficient way of addressing pressing maternal and newborn and other health needs in low and middle-income countries.

Our study focussed on implementers’ actions to enable scale-up. Donors can also foster innovation scale-up. They can allow their implementers flexibility to respond to change, which contributed to the scale-up of sepsis case management in Ethiopia. Donors could support their implementers technically and financially to generate strong evidence to assist governments in deciding which innovations to scale and how to implement them at scale. There would also be considerable value in donors extending funding to implementers through the transition to scale period, thereby enabling them to support governments scale innovations. Donors could also increase the prospects of scale-up through embracing the principles of aid effectiveness. A key approach to achieving this is to ensure they embrace government-led donor coordination mechanisms, and that they require their implementers do so. This is likely to strengthen country ownership, alignment and harmonisation, which were particularly absent in Nigeria, thereby avoiding situations where donors prescribe country health programmes that ‘*disempower government and make them feel like puppets’,* as one of our respondents explained. Rather than simply acting as funding bodies, donor programme officers’ direct involvement in fostering harmonisation among multiple implementers, and brokering relationships with government can also be valuable, which was an approach adopted by the foundation in Ethiopia.

Additionally, recipient governments can take steps to increase the value of externally funded programmes; it is not in their interests for donors to fund short-term projects in their countries with limited prospects of being scaled. Taking ownership of donor-funded innovations and working closely with implementers and donors is likely to help maximise alignment with country priorities, programmes and targets; these were important factors leading to the scale-up of our examples of ‘government-led scale-up’ in Ethiopia and Uttar Pradesh. Indeed, in these settings, with high levels of government ownership, it seems far more likely that donor-funded innovations will be sustained within country health systems in the longer term, in contrast with our example of ‘donor-led scale-up’ in northeast Nigeria. Governments could also strengthen coordination mechanisms to foster information exchange among partners, to help capture and make sense of evidence on innovations to aid decision making around which innovations to scale-up, and to enable government to inform partners of its current and emerging country priorities, programmes and targets to help ensure donor-funded innovations are aligned. Again, it was in Ethiopia where respondents reported this as happening.

This study has a number of limitations. While we provide only a snapshot of the relatively early stages of scale-up of our three case study innovations and were not able to track their longer term progress, our interviews were conducted after the point in time that these innovations had been adopted at scale. Hence our analysis was able to capture the decision-making process leading to their scale-up. A follow-up study is exploring the longer term sustainability of these and other maternal and newborn health innovations in the three settings. Our focus was on successfully scaled innovations; it was beyond the scope of the paper to explore the factors influencing why innovations are not scaled. It was also beyond the scope of the study to quantitatively measure the relative importance of the critical actions identified; our findings do, however, suggest that each action was critical to the scale-up of least one of the case study innovations. We have also simplified highly complex, changing contexts and focussed in detail on only three examples from maternal and newborn health supported by one specific donor. While it is difficult to ascertain how generalisable our findings are to other health innovations and to other settings, it is hoped that the insights presented in this paper will be relevant to other donors and in other settings where project-based funding is common. As the focus of the paper is on why innovations were scaled rather than the experiences of the end users, our data collection drew out the perspectives of decision makers from government, development partner agencies, implementers and frontline workers, but not those of beneficiary communities, who might have views that contrast with those we present in this paper.

## Conclusions

This paper highlights the difficulties of scaling-up project-based donor-funded innovations; the project-based approach tends to imply short-term and often unpredictable funding and leads to limited coordination among multiple actors – factors that undermine efforts to promote scale-up. The paper also shows that while scale-up of donor-funded maternal and newborn health innovations can be difficult, there are approaches that implementers can adopt to increase the prospects of scale-up. The first two were: designing innovations for scale, by ensuring they were effective, simple and acceptable to communities, that they incentivised frontline workers to use them and were adaptable across diverse contexts; and generating a strong base of evidence to influence and inform scale-up including qualitative operational lessons and experiential evidence as well as quantitative impacts data. The other actions were, harnessing the support of powerful individuals; being both prepared for scale-up and responsive to change; ensuring continuity by being part of the transition to scale process; and embracing the aid effectiveness principles of country ownership, alignment and harmonisation. Donors can increase the prospects of scale-up by supporting their implementers to build strong evidence, allowing their implementers flexibility to respond to policy changes, supporting their implementers in the transition to scale process, being active in fostering country ownership, alignment and harmonisation, and increasing the length of time they are prepared to fund projects. Governments can increase the value of externally funded innovations by taking ownership of those innovations, engaging with implementers and donors to help maximise the alignment of innovations with country priorities, programmes and targets and strengthening donor coordination mechanisms to foster better harmonisation among multiple externally funded projects.

## Abbreviations

ASHAs: Accredited Social Health Activists (India)
ETS: Emergency Transport Scheme (Nigeria)
HEWs: Health Extension Workers (Ethiopia)
